# A systematic review on the use of topical hemostats in trauma and emergency surgery

**DOI:** 10.1186/s12893-018-0398-z

**Published:** 2018-08-29

**Authors:** Osvaldo Chiara, Stefania Cimbanassi, Giovanni Bellanova, Massimo Chiarugi, Andrea Mingoli, Giorgio Olivero, Sergio Ribaldi, Gregorio Tugnoli, Silvia Basilicò, Francesca Bindi, Laura Briani, Federica Renzi, Piero Chirletti, Giuseppe Di Grezia, Antonio Martino, Rinaldo Marzaioli, Giuseppe Noschese, Nazario Portolani, Paolo Ruscelli, Mauro Zago, Sebastian Sgardello, Franco Stagnitti, Stefano Miniello

**Affiliations:** 1 General Surgery-Trauma Team, State University of Milano, Niguarda Hospital Milano, Piazza Benefattori dell’Ospedale, 3, 20162 Milan, Italy; 2 General Surgery-Trauma Team, Niguarda Hospital, Milan, Italy; 3 General Surgery, SS Annunziata Hospital, Taranto, Italy; 4 Emergency Surgery Unit, State University of Pisa, Cisanello Hospital, Pisa, Italy; 5 Trauma Surgery Unit-Department of Surgery-Emergency Surgery Department Policlinico Umberto I-Rome, Rome, Italy; 6 Department of Surgical Sciences, State University of Torino, Turin, Italy; 7 Emergency Surgery, Umberto I Hospital, Rome, Italy; 8 Trauma Surgery, Maggiore Hospital, Bologna, Italy; 9 General Surgery and Pancreatic Surgery Unit, State University La Sapienza, Rome, Italy; 10 Emergency Surgery, Ladolfi Hospital, Avellino, Italy; 11 Honorable Chief- Emergency Surgery, Cardarelli Hospital, Naples, Italy; 12 Department of Emergency and Transplant Surgery, State University of Bari, Bari, Italy; 13 Trauma Center, A.O.R.N Cardarelli, Naples, Italy; 14 Department of Clinical and Experimental Sciences-State University of Brescia, Bari, Italy; 15 Department of Emergency Surgery, Ospedali Riuniti, Ancona, Italy; 16 Minimally Invasive Surgery Unit- Policlinico S. Pietro, Ponte San Pietro, Bergamo, Italy; 17 State University of Rome “La Sapienza”-Polo Pontino, Latina, Italy; 18 Department of Surgery, University of Bari, Bari, Italy

**Keywords:** Hemorrhage, Trauma, Emergency surgery, Hemostats, Fibrin adhesives, Sealants, Mechanical hemostats, Hemostatic dressings, Systematic review

## Abstract

**Background:**

A wide variety of hemostats are available as adjunctive measures to improve hemostasis during surgical procedures if residual bleeding persists despite correct application of conventional methods for hemorrhage control. Some are considered active agents, since they contain fibrinogen and thrombin and actively participate at the end of the coagulation cascade to form a fibrin clot, whereas others to be effective require an intact coagulation system. The aim of this study is to provide an evidence-based approach to correctly select the available agents to help physicians to use the most appropriate hemostat according to the clinical setting, surgical problem and patient’s coagulation status.

**Methods:**

The literature from 2000 to 2016 was systematically screened according to PRISMA [Preferred Reporting Items for Systematic Reviews and Meta-Analyses] protocol. Sixty-six articles were reviewed by a panel of experts to assign grade of recommendation (GoR) and level of evidence (LoE) using the GRADE [Grading of Recommendations Assessment, Development and Evaluation] system, and a national meeting was held.

**Results:**

Fibrin adhesives, in liquid form (fibrin glues) or with stiff collagen fleece (fibrin patch) are effective in the presence of spontaneous or drug-induced coagulation disorders. Mechanical hemostats should be preferred in patients who have an intact coagulation system. Sealants are effective, irrespective of patient’s coagulation status, to improve control of residual oozing. Hemostatic dressings represent a valuable option in case of external hemorrhage at junctional sites or when tourniquets are impractical or ineffective.

**Conclusions:**

Local hemostatic agents are dissimilar products with different indications. A knowledge of the properties of each single agent should be in the armamentarium of acute care surgeons in order to select the appropriate product in different clinical conditions.

## Background

A wide variety of hemostats are available as adjunctive measures to improve hemostasis during surgical procedures if residual bleeding persists despite correct application of conventional methods for hemorrhage control.

Some are considered active agents, since containing fibrinogen and thrombin and actively participating at the end of the coagulation cascade to form a fibrin clot [1–4]. These agents can be used effectively in patients with spontaneous or drug-induced coagulation disorders. They are available in liquid flowable form (fibrin glues) [1–9] or in association with stiff collagen fleece (fibrin patch) [10–14]. These agents are known as *adhesive hemostats* because of their hemostatic and tissue sealing action [15–33].

Others, including porcine gelatin [34], oxidized cellulose [35, 36], bovine collagen [37], and plant-derived polysaccharides spheres [38, 39], are known as *mechanical hemostats*, by providing platelet activation and aggregation and forming a matrix at the site of bleeding, which allows clotting to occur. Since they are not biologically active, relying on patient’s own fibrin production to achieve hemostasis, these agents are considered passive hemostats and are only appropriate for patients who have an intact coagulation system.

*Sealants* are low viscosity liquids that polymerize forming a solid film that connects a-traumatically the tissue surfaces [40–53]. This property makes these agents effective both as sealants and as hemostats. They can be divided in synthetic (cyanoacrylate and polyethylene glycol-PEG sealants) [42–51] and semisynthetic (glutaraldheide albumin-derived sealants) [52, 53].

*Hemostatic dressings* represent a valuable option to achieve hemostasis in the case of external hemorrhage at junctional sites (axilla, groin), when the use of a tourniquet is ineffective or impractical. [54–64] They are classified as either factor concentrators (zeolite) [57, 58], procoagulants (kaolin) [59], and mucoadhesives (chitin) [60, 61]. They are available as granules/powders or bandages [62] and are indicated for external use only.

The aim of this study is to provide an evidence-based approach to correctly select the available agents, in order to help physicians to use the most appropriate hemostat in accordance with clinical setting, surgical problem and patient’s coagulation status.

## Methods

The Organizing Committee (O.C., S.C.) was established to plan a National Meeting on the use of topical hemostats in emergency surgery and trauma. The meeting was conducted according to “The Methodological Manual-How to Organize a Consensus Conference”, edited by the Higher Health Institute [65]. The meeting was powered by the Italian Society of Emergency Surgery and Trauma (SICUT) and endorsed by the World Society of Emergency Surgery (WSES) and its Italian Chapter (WSES.it). The representatives of the executive board of both societies were asked to participate. A representative of each of the following companies (ASSUT, Baxter, B-Braun, Takeda, Johnson &Johnson, Medtronic, Medica, Valeggia, Medical, SVAS) attended the meeting as auditors. The organizing committee selected a scientific board (SB, 4 members) and a national panel of experts (NPE, 5 members). The organizing committee and SB selected the following main topics:

1. Adhesives
  Liquid fibrin adhesives
  Fibrin patch
2. Mechanical Hemostats
3. Sealants
4. Hemostatic Dressings (mineral and polysaccharides)

Each panelist was asked to answer the following key questions and specific sub-questions pertaining to the assigned topic:

1. Is the hemostat effective in the presence of coagulation disorders?
2. Which type of hemorrhage (i.e. oozing, spurting, mild, moderate) is best managed by the agent?
3. Is the agent effective to control fluid leakages (biliary, pancreatic, urinary)?
4. Is the agent effective to control air leak?
5. Is the agent effective to protect vascular anastomosis?
6. Is the agent effective to protect intestinal anastomosis?
7. Which are the possible cautions against use of the single agent?

A systematic review of the literature from 2000 to 2016 was undertaken by a medical reference librarian in May 2016. An investigator (S.C.) created a preliminary search strategy by selecting the following key words: emergency surgery, trauma, hemorrhage, hemostasis, hemostatic agents, biliary fistula, urinary fistula, pancreatic fistula, anastomosis, air leak. Searches were conducted incorporating novel terms when relevant citations were found using the following database: MEDLINE, PubMed, EMBASE, Scopus, and Cochrane Database of Systematic Reviews. The investigator (S.C.) screened titles and abstracts selecting studies according to PRISMA statements [66]. The following types of articles were included: (I) prospective randomized controlled trials; (II) observational studies in which data were collected prospectively; (III) retrospective analyses based on clearly reliable data; (IV) systematic reviews of literature; (V) meta-analyses; and (VI) relevant case series: all articles eligible for evaluation were divided according to the selected topics and sent for further evaluation to the five national experts. They were asked to assign levels of evidence (LoE) and grades of recommendations (GoR) based on the Grading of Recommendations, Assessment, Development and Evaluation (GRADE) hierarchy criteria (Table 1) [67].

**Table 1:** Grading of Recommendation from Guyatt et al. [67] (GRADE)

1A. Strong recommendation, high-quality evidence	Benefits clearly outweigh risks and burdens, or vice versa	RCTs without important limitations or overwhelming evidence from observational studies	Strong recommendation, applies to most patients in most circumstances without reservation
1B. Strong recommendation, moderate-quality evidence	Benefits clearly outweigh risk and burdens, or vice versa	RCTs with important limitations (inconsistent results, methodological flaws, indirect analyses or imprecise conclusions) or exceptionally strong evidence from observational studies	Strong recommendation, applies to most patients in most circumstances without reservation
1C. Strong recommendation, low-quality or very low-quality evidence	Benefits clearly outweigh risk and burdens, and vice versa	Observational studies or case series	Strong recommendation but subject to change when higher-quality evidence becomes available
2A. Weak recommendation, moderate-quality evidence	Benefits closely balanced with risks and burdens	RCTs without important limitations or overwhelming evidence from observational studies	Weak recommendation, best action may differ depending on the patient, treatment circumstances, or social values
2B. Weak recommendation, moderate-quality evidence	Benefits closely balanced with risks and burdens	RCTs with important limitations (inconsistent results, methodological flaws, indirect analyses or imprecise conclusions) or exceptionally strong evidence from observational studies	Weak recommendation, best action may differ depending on the patient, treatment circumstances, or social values
2C. Weak recommendation, low-quality or very low-quality evidence	Uncertainty in the estimates of benefits, risks, and burdens; benefits, risks, and burdens may be closely balanced	Observational studies or case series	Very weak recommendation, alternative treatments may be equally reasonable and merit consideration

On May 15, 2017, a meeting was held involving the organizing committee, NPE, SB, and the representatives of the scientific societies to discuss topics and define statements to be presented during the 45th SICUT Congress, held in Naples in October 2017. For each topic, a 35 min-session was held with a presentation of hemostatic agent characteristics, a literature review by a member of the NPE, and a discussion with the representatives of the Societies. It was all recorded for later analysis and subsequent manuscript preparation.

## Results

**Fig. 1:**
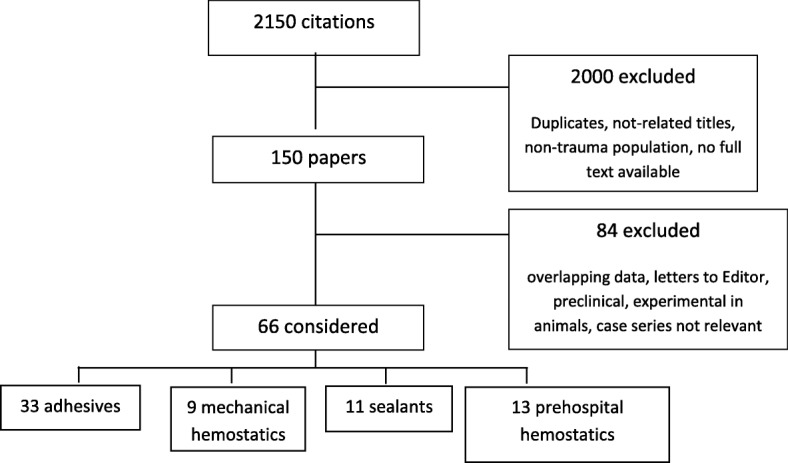
Bibliography search (PRISMA). This figure represents the methodology applied to screen abstract and papers, in order to identify relevant papers to be used for manuscript preparation

The database searches identified 2150 citations (Fig. 1) By removing duplicates, titles not related to the topic, case reports, articles on non-trauma patients, articles on pediatric trauma, articles on penetrating trauma, and articles where a full text was unavailable, 2000 citations were excluded. Of the remaining 150 citations, 84 were excluded due to overlapping data or because they were letters to the editor. The resulting 66 papers were divided according to each topic: 33 for topic 1, 9 for topic 2, 11 for topic 3, 13 for topic 4.

### General considerations

*Hemostats, irrespective of their nature, are not intended as a substitute for a sound surgical technique and proper application of ligatures or other conventional procedures for hemostasis (1 A).*

#### Scientific foundation

Surgical bleeding can be associated with an increased risk of mortality and morbidity across all surgical areas. In particular, bleeding complications arise in nearly 30% of surgical procedures [2]. Moreover, excessive bleeding often leads to increased mortality-morbidity, longer hospital stays, and increased healthcare costs. Hemostasis can be achieved with conventional techniques such as manual pressure and ligature; however, these can be ineffective in controlling bleeding from complex injuries and in less accessible areas. Energy-based methods, such as electrocauterization or laser cauterization can also be used; however, they create charred areas and necrotic tissue, which increase the likelihood of infection.

Topical hemostats encompass a wide variety of products which may help bleeding control, mostly in challenging surgical settings or if patients are affected by congenital or acquired coagulation disorders. Each category of topical hemostat presents its own properties, which makes it eligible for use in different circumstances.

Notwithstanding the contribution of these agents, irrespective of their nature, composition and mechanism of action, they should not be intended as a substitute for a sound surgical technique.

The classification of different agents, their mechanism of action, advantages and caution against use are depicted in Table 2.

**Table 2:** Hem Adjunctive measure to control residual mild or moderate bleeding (oozing and/or spurting) also in coagulation disorders

Category	Commercial name	Action	When to use	Cautions
Liquid adhesives	*Fibrinogen + thrombin:* Tisseel, Evicel, Beriplast, Vitagel, Cryoseal, Evarrest, Crosseal, Quixil *Human Thrombin:* Evithromb, Thrombin-JMI, Rechothromb *+ Bovine gelatin* Floseal *+ Porcine gelatin* Surgiflo	Fibrinogen and thrombin mixed at the site of application to form clot.	Adjunctive measure to control residual mild or moderate bleeding (oozing and/or spurting). Effective irrespective of patient’s coagulation status. Weak sealant effect	Do not use in individuals known to react to human blood products. Increased risk of thrombosis with bovine thrombin. Avoid intravascular administration because of thromboembolic risk. Avoid tranexamic acid containing products if CSF leak because of neurotoxicity. Gelatine granules can swell up to 20%
Adhesives with mechanical support	*Fibrinogen + thrombin + equine collagen patch* TachoSil	The patch facilitates compression on bleeding site	Adjunctive measure to control residual severe bleeding because the patch prevents the streaming effect of blood. Weak sealant effect	
Mechanical hemostats	-*Oxidized Cellulose* Surgicel (fibrillar, original) Nu-Knit (fibrillar, original) *-Collagen (microfibrillar- MFC)* Avitene, Helistat/Helitene, Instat/Ultrafoam *-Porcine gelatin* Gelfoam (flour, sponge, plus) Surgifoam *-Polysaccharides spheres* Arista AH	These agents can be used only in patients with normal coagulation system. They form a tri-dimensional matrix at the site of bleeding, which allows clotting to occur. Provides physical matrix for clotting initiation; low pH contributes to platelet activation Platelet adherence and activation Concentrates platelets and clotting factors	Very good handling characteristics; does not stick to instruments; typically dissolves in 2–6 weeks; low pH has antimicrobial effect; effective on residual oozing No significant swelling Effective despite heparinization Nonantigenic; neutral pH allows use with biological agents; effective on oozing and bleeding from bone Rapidly absorbed; effective on residual mild bleeding if conventional method impractical	Not effective in coagulation disorders It inhibits thrombin because of low pH; may induce inflammation for foreign body reaction; caution if used in narrowed spaces because it can swell. Less effective if thrombocytopenia; may bind to neural structures; avoid if blood-saving device used as MFC can pass through the filters Caution if used in confined spaces as it swells; remove the excess after obtained hemostasis; may embolize in intravascular compartments Caution in diabetic patients; Risk of embolism if accidentally injected or placed within a blood vessel.
Hemostatic dressings	*Zeolite* QuickClot ACS+ *Smectite* WoundStat *Kaolin* Quick Clot Combat Gauze (CG) *Chitin/chitosan* HemCon Celox (CE) (granules, gauze)	Molecular sieves absorb water at site of wound and increase concentration of clotting factors, platelets, RBCs. Procoagulant agents which enhances coagulation by providing a high local concentration of coagulation factors. Muco-adhesive agent with direct electrostatic interaction between negatively charged cell membranes of the erythrocytes and positively charged chitosan	Control of significant hemorrhage in anatomic areas where tourniquets cannot be applied and where sustained direct pressure alone is not feasible (neck, groin, axilla) Bleeding wounds on the field Bleeding wounds on the field. Can be poured into wound and conform to it. Easily portable Removal much easier than other granules/powder agents	Exothermic reaction with risk of burn at the site of application for both the products Not biodegradable, must be completely removed before definitive repair Allergy to crustaceans. Eccessive stiffness

ostats: mechanisms of action, advantages and cautions against use

### Adhesives

Adhesives are active agents which participate at the final step of coagulation cascade to form a fibrin clot. They are constituted by two components: human purified fibrinogen and/or thrombin. The two components usually contain added plasmatic proteins such as factor XIII, fibronectin, and an anti-fibrinolytic agent like aprotinin or tranexamic acid [7]. Nowadays, aprotinin-free and tranexamic acid-free formulations are available [3, 5] reducing the risk of side effects, such as aprotinin-associated hypersensitivity reactions, and tranexamic acid-associated neurotoxicity [3, 5, 20–23]. Several adhesives are available as liquid formulations (Evicel®, Crosseal™, Quixil®, Floseal®, Surgiflo®, Tisseel®, Beriplast P®) or as medicated sponge/patch (Tachosil®, Evarrest®). Once applied irrespective of their formulation, their mechanism of action is the same: thrombin cleaves the fibrinogen into fibrin monomers to form a soluble matrix. Thrombin also activates factor XIII to factor XIIIa, which covalently crosslinks the soluble fibrin matrix to form a stable clot. Both these steps require the presence of calcium ions [1]. Adhesives do not induce either inflammation response or tissue necrosis, and they are metabolized by fibrinolysis and phagocytosis in the same way as endogenous fibrin [1].

### Liquid fibrin adhesives

1. *Liquid fibrin adhesives can be used as an adjunctive measure, after sound surgical hemostasis, to improve residual mild or moderate bleeding (oozing and/or spurting) (1B)*
2. *Liquid fibrin adhesives are effective also in cases of coagulation disorder, however, because they are expensive, their use should be preferred in those patients with spontaneous or drug-related coagulation disorders (1A)*
3. *Fluid fibrin adhesives are more effective to control residual bleeding on huge and regular raw surfaces (2C)*
4. *Foam fibrin adhesives are more effective to control residual bleeding in irregular and deep raw surfaces (2C)*
5. *Intravascular administration of liquid fibrin adhesives must be avoided because the thromboembolic risk is high (1 B)*
6. *Liquid fibrin adhesives are effective to control residual bleeding on vascular anastomosis (1B)*
7. *Liquid fibrin adhesives containing tranexamic acid must not be used if cerebrospinal fluid leakage or dura mater tear are present, because of neurotoxicity (1A)*
8. *Liquid fibrin adhesives are not effective in preventing pancreatic, biliary, urinary, intestinal and air leakages. Therefore, their routine use should not be recommended (1B)*

#### Scientific foundation

Although all liquid fibrin products contain fibrinogen and/or thrombin, they differ from each other in several respects, including composition and ease of use. One aspect is the speed of hemostasis, the characteristics of fibrin clot and the agent’s safety profile [1]. In particular, a higher fibrinogen concentration tends to produce a stronger, but slow forming clot, whereas higher thrombin concentrations result in rapid clot formation, but the clot formed is not as strong.

Liquid fibrin adhesives are applied by means of a single or double syringe system, which allows the application through a blunt needle or spray tips. The spray tip is particularly useful when the surgical setting requires a large and uniform deposition of the product [1, 7]. Fluid formulations are more effective if the raw surface is huge and regular, whilst foam products seem to be preferred if the bleeding surface is deep in the parenchyma and irregular, because of better adherence to tissue of this physical form [2], due to its tendency to expand.

Safety concerns include the fact that any product containing thrombin should never be injected intravascularly because this administration may be associated with thrombosis, hypotension and death. Moreover, a risk of air embolism is present with the use of gas driven sprayers, particularly when the adhesive is applied at higher pressures (> 20–25 psi) against manufacturer recommendations, or at shorter distances (< 10–15 cm) [7].

Liquid fibrin products, when conventional hemostatic methods are ineffective or impractical, can be used to control residual mild or moderate bleeding in several surgical settings.

Craig and coworkers [5], in a randomized, multicenter, active-controlled study evaluated the efficacy and the effectiveness of liquid fibrin adhesive, in comparison to mechanical hemostats, to control mild to moderate bleeding on 124 patients undergoing retroperitoneal and intraperitoneal procedures at 10 min after randomization. Overall, a higher percentage of patients who received liquid fibrin adhesive achieved hemostasis within 10 min compared with those who received the mechanical hemostat (RR 1.16, 95% CI, 1.02–1.35; *p* < .05). In particular, 100% of patients managed with liquid fibrin adhesive for mild bleeding achieved hemostasis within 10 min, versus 88.9% of subjects receiving mechanical hemostat (*p* < .002). Moreover, a higher percentage of patients who received liquid fibrin adhesive to treat moderate bleeding achieved hemostasis within 10 min, compared with those who received mechanical hemostat (87% vs 73.1%, respectively, *p* < .05).

Mimicking the end steps of the coagulation cascade, supplying exogenous fibrinogen and/or thrombin, liquid fibrin adhesives are particularly effective in achieving hemostasis in congenital or acquired bleeding disorders (e.g. hemophilia patients, patients receiving antiplatelet or anticoagulation therapies undergoing urgent surgical procedure) [1, 3, 9].

There is evidence that bleeding control and postoperative blood transfusion requirements in patients with hemophilia undergoing major orthopedic procedures, such as total knee and total hip replacement, improve if liquid fibrin adhesives are used [1].

Liquid fibrin adhesives seem to be effective in reducing the time to achieve hemostasis in post-cardiopulmonary bypass coagulopathy patients, in decreasing the amount of intraoperative blood loss and of blood product requirements [1, 3].

Notwithstanding these advantages, the cost of the fibrin sealants, ranging from $50 to $600 [7], is not negligible, and may also be important in determining therapeutic choices.

One cost-effectiveness analysis [1] conducted in the UK on patients undergoing total knee replacement estimated that use of a 5 mL dose of Quixil® in addition to conventional hemostatic methods was cost saving compared to conventional methods alone, and reduced the expected cost by £296 per procedure. However, use of a 10 mL dose increased the expected cost by £94 per procedure. For this reason, the use of liquid fibrin adhesive should be titrated on selected cases.

Several studies are available [3, 8, 9] pertaining to the effectiveness of liquid fibrin adhesives in controlling residual bleeding after vascular surgery.

In a prospective randomized controlled trial, Chalmers et al. [3], demonstrated the effectiveness of Evicel®, a tranexamic-acid free adhesive, in achieving hemostasis on polytetrafluoroethylene (PTFE) arterial anastomosis within 4 min, in comparison with manual compression. A significantly higher percentage of patients who received fibrin adhesive achieved hemostasis within 4 min compared with those who received manual compression alone (85% vs 39%, *p* < .001), irrespective of the type of artery treated.

Taylor et al. [8] in a single-blinded randomized prospective multicenter trial investigated the safety and effectiveness of Beriplast P® versus thrombin-soaked gelatin sponge (TSG) for suture needle or needle-hole bleeding from PTFE artery grafts. On 201 subjects, 63% of patients with fibrin adhesive achieved hemostasis within 4 min, compared with 40% in the TSG group (*p* = .0018). In the treatment group, time to hemostasis was shorter (median, 40 min, *p* = .008), and blood loss was less (median, 15 min, *p* = .005).

Echave and coworkers [2], in a systematic review, analyzed available data on the effectiveness of human gelatin-thrombin matrix sealant in different surgical fields (cardiovascular, orthopedic, otorynolaryngology, urology). All the 27 studies included in the review demonstrated that the gelatin-thrombin matrix was associated with a significantly higher rate of successful hemostasis and a shorter time to achieve it (*p* < .001 for both) in comparison with other alternatives when conventional methods failed.

Saha and coworkers [9] assessed the effectiveness of TISSEEL® for the treatment of anastomotic suture-hole bleeding in expanded PTFE grafts. In a prospective randomized controlled study on 140 patients who experienced suture-line bleeding that required treatment after completion of anastomotic suturing, the use of TISSEEL® brought about hemostasis within 4 min in 62.9% of subjects, in comparison with 31.4% of controls (*p* < .001). Similarly, hemostasis rates in the subgroup of patients on antiplatelet therapy were 64.7% in the treatment group and 28.2% in controls, respectively (*p* < .001).

Liquid fibrin adhesives are also effective as sealants, an adjunctive measure which improves the watertight closure of the dural suture line thus preventing cerebrospinal fluid leak (CSF) [20–23]. Nevertheless, cautions in using fibrin adhesive containing tranexamic acid in a neurosurgical setting is necessary, because of its neurotoxicity. In fact, some evidence from preclinical studies has shown that tranexamic acid induced hyperexcitability and convulsions, probably caused by blocking GABA-mediated inhibition in the central nervous system [1, 20–23].

No incontrovertible evidence regarding the effectiveness of liquid fibrin adhesives in preventing pancreatic, biliary, intestinal and air leakages is available.

Chen et al. [27] in a recent systematic revision of the literature about the use of fibrin-based products in preventing postoperative pancreatic fistula (POPF) following pancreatic surgery stated that the use of fibrin adhesives showed no significant advantages in terms of the reduction of POPF and that they do not decrease postoperative mortality, morbidity, or reoperation rates, discouraging the routine application of these products. Conflicting results are available on the biliostatic effects of fibrin adhesives [1, 6, 7, 16, 17]. In a prospective randomized controlled trial on 121 patients who underwent liver resection, Schwartz et al. [6] compared the effectiveness of Crosseal® with that of different products, including various collagens, cellulose, gelatin and thrombin for the control of bile loss. The authors found that the percentage of patients who needed reoperation for biliary complications was lower in the Crosseal® group than in the other groups (17.2% vs 36.5%, respectively; 2-sided *p* = .002). No significant difference between them was recorded about the duration of postoperative bilious drainage, the number of patients suffering from bile leak, the amount and duration of drainage, although differences always favored the Crosseal® group.

Figueras et al. [17] in a prospective randomized single-center trial on 300 hepatectomy cases found no difference in the incidence of biliary fistula between patients treated with liquid fibrin adhesive and controls (10% vs 11%, respectively). These results were confirmed also by Erdogan and coworkers [16] in a systematic review of the literature; the conclusion drawn was that fibrin adhesives cannot prevent leakages from the bile duct on the resection surface with certainty.

Papers regarding the use of fibrin adhesives as an adjunctive measure to prevent leakage of intestinal anastomosis are lacking with conflicting results [1]. Some suggest possible benefits in ileal and gastric/bariatric anastomoses, while others did not find convincing results about fibrin adhesive as an external coating of colonic anastomoses [25, 26].

In thoracic settings, the prophylactic use of liquid fibrin adhesive to prevent air leak does not seem useful if air control is good at the end of the procedure. Moreover, if fibrin adhesives are applied to control persistent air leak at the end of conventional surgical manoeuvers, they do not significantly reduce the time of chest tube removal [1].

### Fibrin patch

1. *In addition to the previous ones, fibrin patch is effective also in the presence of ongoing severe hemorrhage, since it prevents the “streaming effect” of blood (1B)*
2. *Fibrin patch is effective to control residual bleeding on vascular anastomosis (1C)*
3. *Fibrin patch has lack effect in preventing air leakage if complete sealing has been achieved using standard techniques, therefore, its prophylactic use should not be recommended (1B)*
4. *Weak evidences are available about the role of fibrin patch in preventing pancreatic, biliary and urinary leakages. Therefore, their routinely use should not be recommended (1B)*

#### Scientific foundation

In addition to fluid texture, fibrin adhesives are also available as a patch (TachoSil®, Takeda; PGA-felt®, Neovel; Fibrin Pad®, Omrix Biopharmaceuticals). These products have different compositions but the same advantages compared with fluid formulations, that are represented by the mechanical support offered by collagen or oxidized cellulose/polyglactin 910 matrix, bounding coagulation factors, which allow a better adherence to bleeding tissues, irrespective of brisk bleeding, preventing the “streaming effect” observed with fluid adhesives [10–15].

TachoSil® is a ready-to-use fixed combination hemostatic agent, consisting of a white honeycomb-like equine collagen patch coated with coagulation factors, human fibrinogen, and human thrombin on one side (yellow colored with riboflavin for orientation).

Fibrin Pad® and PGA-felt® are absorbable hemostats composed of well-characterized material (polyglactin [PG 910], oxidized regenerated cellulose, thrombin and fibrinogen) designed to be effective in a variety of tissue types and across a spectrum of bleeding intensities [10].

These products have all been proven to be effective in rapidly achieving hemostasis in severe bleeding during abdominal, retroperitoneal and pelvic surgical procedures, compared to the standard of care [10–12]. TachoSil® has also been employed as reinforcement for the sutured anastomosis of the portal vein and has been shown to be useful in the repair of hepatic artery pseudoaneurism [15]. It has also been proven to be effective in cardiovascular surgery. Maisano et al. [13] in a randomized, parallel-group, multicenter trial demonstrated that TachoSil® was significantly superior to standard hemostatic fleece in controlling bleeding after insufficient primary hemostasis, with 75% of patients in the TachoSil® group achieving hemostasis at 3 min compared with only 33% of the standard treatment group (*p* < .001).

On the other hand, the effectiveness of fibrin patch in preventing air leaks after thoracic surgery procedures, and of pancreatic and bile leaks after resective interventions is a matter of discussion.

Anegg and coworkers [31] in a randomized study on 173 patients who had undergone a lobectomy or segmentectomy and were suffering from persistent air leakage demonstrated a significant reduction of intraoperative air leak after application of TachoSil® (153.32 ml/min vs 251.04 ml/min, *p* = .009) in comparison to the control group. This reduction in air leakage volume resulted in a significant shortening of the drainage period (5.1 days vs 6.3 days, respectively; *p* = .002) and time to discharge (6.2 days vs 7.7 days, respectively; *p* = .01).

On the other hand, Belda-Sanchis et al. [32] in a systematic literature review demonstrated that if a complete sealing has been reached using standard techniques, the prophylactic use of fibrin patch to prevent postoperative air leaks is not justified.

Considerations for the use of fibrin patch in the prevention of bile and pancreatic leakages is controversial with a plethora of studies supporting both sides of the argument [15].

TachoSil® has been found to be more effective in decreasing bile leak after adult liver transplantation than fibrin glue applicated on the cut surface (6.25% vs 43.75%, respectively; *p* = .03) [15].

On the other hand, Kobayashi et al. [18], in a multicenter randomized controlled trial involving 786 patients who had undergone hepatectomy, compared the effectiveness of PGA felt® (PGA-FS) versus fibrinogen-based collagen fleece (CF) at the liver cut surface for the prevention of bile leakage. They observed no significant differences between the two groups, recording a bile leakage rate of 4.1% in PGA-FS vs 5.1% in CF, respectively; *p* = .51). Due to the heterogenicity of available evidence, the routine use of fibrin patch in this setting should not be recommended.

Conflicting data are also available on the effectiveness of fibrin patch in preventing pancreatic fistula after major pancreatic resections [27–29]. Montorsi and coll [27] in a prospective, open, randomized study on 275 patients requiring distal pancreatectomy (DP) compared the fistula rate between patients in whom TachoSil® was applied on the pancreatic stump and those who did not received the fibrin patch. The authors observed no significant difference between the groups (62% treatment group vs 68% controls, respectively; *p* = .276). A significantly lower amylase concentration in the drainage was found in the TachoSil® group on postoperative day 1, but increasing on day 3 and 5, becoming similar to that recorded in the control group. Marangos et al. [28] in a retrospective analysis of prospectively collected data on 121 patients who had undergone DP found that covering the pancreatic stump with TachoSil® did not affect either the occurrence of POPF or duration of postoperative hospital stay. Finally, Cheng et al. [29] in a Cochrane revision of the available literature stated that considering the lack of effect on the prevention of POPF, high costs, and the potential harm for endocrine pancreatic function, fibrin patch should not be recommended routinely for people undergoing pancreatic surgery.

Table 3 summarized the referral papers for topic 1.

**Table 3:** Reviewed papers for adhesives

	Year	Design	Comments	GoR-LoE
Annegg U et al. [31].	2007	Ranzomized, single-center	The use of TachoSil® after pulmonary resection resulted in reduction of air leak with significant shortening of time of tube removal and of hospital length of stay	1C
Belda-Sanchis et al. [32]	2010	Cochrane review	Surgical sealants have some beneficial effects in reducing postoperative air leaks, but their systematic use cannot be recommended at the moment	1A
Briceno et al. [24]	2010	Prospective controlled	The fibrin sealant after major liver resection was effective for decreasing drainage volume; postoperative blood transfusion requirements; moderate to severe postoperative complications and mean hospital stay	1C
Chalmers et al. [3]	2010	Prospective randomized	Tranexamic acid-free fibrin sealant is safe, and significantly shortened the time to haemostasis in vascular procedures using PTFE	1B
Chapman et al. [4]	2007	Phase 3, randomized, double-blind	Recombinant thrombin has comparable efficacy, a similar safety profile, and is considerably less immunogenic than bovine thrombin when used for surgical hemostasis	1C
Cheng et al. [29]	2016	Cochrane review	Considering the lack of effect on prevention of POPF, high costs, and the potential harms for endocrine pancreatic function, fibrin sealants should not be recommended routinely for people undergoing pancreatic surgery	1A
Colombo et al. [14]	2014	Systematic review	TachoSil® has a role as a supportive measure to improve hemostasis and promote tissue sealing when standard techniques are insufficient	1B
Cormio et al. [33]	2012	Prospective randomized	TachoSil sealed tubeless PCNL does not reduce pain and analgesic requirements, but it significantly reduces urinary leakage and postoperative hospital stay	2A
De Boer et al. [19]	2012	Systematic review	Fibrin sealants can be effective as an adjunct to achieve hemostasis during liver resections. However, considering lack of evidence on the efficacy of fibrin sealants in reducing postoperative resection surface-related complications, routine use of fibrin sealants in liver surgery cannot be recommended.	1B
Dhillon et al. [1]	2011	Systematic review	Liquid fibrin sealants are effective as adjunctive measure to improve hemostasis	1B
Echave et al. [2]	2014	Systematic review	Floseal® showed improvement over other hemostatics agents in achieving hemostatis and reducing blood loss	1B
Erdogan et al. [16]	2007	Systematic review	There is no clear proof of the biliostatic efficacy of topical hemostatic agents used after liver resection on the resection surface	1B
Esposito et al. [23].	2016	Systematic review	Fibrin sealants provide a higher rate of intraoperative watertight closure of dura suture line and they may be effective in preventing cerebrospinal fluid leaks with an acceptable safety profile	1B
Figueras et al. [17]	2007	Prospective randomized	Application of fibrin sealants in the raw surface after hepatectomy does not seem justified	1C
Filosso et al. [30]	2013	Prospective randomized	TachoSil® was superior to standard stapling and suturing aerostatic techniques in reducing postoperative air leaks in patients undergoing redo thoracic surgery.	1B
Fisher et al. [5]	2011	Randomized, multicenter	Tranexamic acid- and aprotinin-free fibrin sealant is safe and effective for achieving hemostasis in soft tissue during elective retroperitoneal or intra-abdominal surgery.	1B
Fisher et al. [10]	2013	Prospective randomized	Fibrin pad is superior to absorbable hemostat in soft-tissue bleeding control and is safe and effective for rapidly and reliably achieving hemostasis	1B
Genyk et al. [12]	2016	Multicenter randomized open-lable	The FSP (TachoSil®) was safe and superior to ORCG (Surgicel Original) for achieving hemostasis in patients undergoing hepatic resection	1B
Green et al. [22]	2015	Multicenter, prospective randomized	Fibrin sealant is effective as an adjunct to dural sutures to provide watertight closure of the dura mater in neurosurgery	1C
Jankowitz et al. [21]	2009	Retrospective	The use of fibrin glue for dural repair did not significantly decrease the incidence of a persistent cerebrospinal fluid leak	2A
Kobayashi et al. [18]	2016	Multicenter randomized	Fibrin sealant with polyglicolic acid compared with collagen fleece did not reduce biliary leakage and hemorrhage	1C
Koea et al. [11]	2015	Randomized controlled	The FP is safe and superior to SoC for controlling challenging severe soft-tissue bleeding encountered during intra-abdominal and thoracic surgical procedures	1C
Maisano et al. [13]	2009	Randomized controlled	TachoSil® was significantly superior to standard haemostatic fleece material in obtaining effective and fast intra-operative haemostasis in cardiovascular surgical procedures. TachoSil was safe and well tolerated.	1B
Montorsi et al. [27]	2012	Multicenter randomized controlled	TachoSil® had no significant effect on the rate of POPF, although there was a significant reduction of amylase level in drainage fluid on postoperative day 1.	1B
Marangos et al. [26]	2011	Retrospective	The application of the TachoSil® patch did not affect either occurrence of POPF or duration of postoperative hospital stay. Routine use of TachoSil® patch to prevent pancreatic fistulas does not provide clinically significant benefit.	1C
Perussi Biscola et al. [20]	2017	Systematic review	The new heterologous fibrin sealant from snake venom represents a consistent alternative to biological sealants, since they may avoid transmission of infectious diseases	1C
Pommergaard et al. [26]	2013	Systematic review	Liquid fibrin adhesive should be beneficial to prevent leak of ileal anastomoses in gastric and bariatric surgery	1C
Saha et al. [9]	2012	Prospective randomized	Fibrin sealant is safe and its efficacy is superior to manual compression for hemostasis in patients with vascular ePTFE graft	1C
Schwartz et al. [6]	2004	Prospective randomized controlled	Crosseal fibrin sealant significantly reduces the time of hemostasis following liver resection in comparison with the standard topical hemostatic agents	1C
Simo et al. [15]	2012	Systematic review	Application of TachoSil® in hepatobiliary and pancreatic surgery has proven effectiveness in hemostasis and as tissue sealant	1C
Spotniz et al. [7]	2012	Systematic review	Fibrin sealants have multiple new uses that should result in further improvement in patient care	1C
Taylor et al.[8]	2003	Randomized prospective multicenter	Fibrin sealants are more effective than thrombin-soaked gelatin sponge for achieving hemostasis of needle or suture hole bleeding from PTFE femoral artery grafts.	1B
Vakalopoulos et al. [25]	2012	Systematic review	Fibrin sealant seem not be necessary as external coating of colonic anastomoses to prevent leakage	1C

### Mechanical hemostats

1. *Mechanical hemostats are only appropriate for patients with an intact coagulation system (1A)*
2. *These agents can be used, in patients without coagulation disorders, to improve control of residual oozing (1A)*
3. *Non-regenerated oxidized cellulose-based topical hemostats are more effective, but unhandy compared with regenerated ones (1B)*
4. *The minimum effective amount of oxidized cellulose-based topical hemostatic should be used, and excess material not contributing to hemostasis should be removed, because of the risk of foreign body reactions (1A)*
5. *The use of oxidized cellulose-based topical hemostats is not indicated to control biliary, urinary, pancreatic, and air leakages (1A)*
6. *Oxidized cellulose-based topical hemostats should not be left in place in proximity to nerves, ureters, intestinal and vascular structures, because of the risk of ischemia due to compression or local inflammatory reaction (1C)*
7. *Oxidized cellulose-based topical hemostats can be used as a complementary adjunct to packing to control bleeding from parenchymal injuries (2C)*
8. *Gelatins may induce, due to their swelling, a compression of other nearby anatomic structures; thus the minimal amount of agent to achieve hemostasis should be used (1C)*
9. *Caution should be used with bovine collagen-based products during surgical procedures that involve blood-saving devices, because the agents can pass through the filters (1C)*
10. *Caution should be used with plant-derived polysaccharides in diabetic patients, because amounts greater than 50 g could affect the glucose load (2A)*

#### Scientific foundation

Mechanical hemostats include oxidized cellulose, gelatin, collagen and plant-derived absorbable products which promote platelet activation and aggregation when directly applied to the bleeding surface, absorbing fluid several times their own weight. This mechanism of action produces a matrix at the site of bleeding, activating the extrinsic clotting pathway which allows the clotting to occur. Mechanical hemostats rely on fibrin production to achieve hemostasis; therefore, these agents are only appropriate for patients who have an intact coagulation cascade [35]. Some studies [35, 36] demonstrated that mechanical hemostats failed to induce platelet activation in the absence of plasma constituents, especially factors VIII and XII. In patients with a functional intact coagulation system these agents can be used as first-line agents because of their immediate availability and as they are highly cost-effective [35–38]. They represent a useful adjunct, with a direct pressure on the bleeding site, to control minimal residual hemorrhage [35].

Oxidized cellulose (OC) (Surgicel®, Surgicel Fibrillar®, Surgicel Nu-Knit®, Johnson & Johnson) is well known and accepted because of its ease of use, favorable biocompatibility and bactericidal properties. Cellulose is a homopolysaccharide which is created through polymerization of glucopyranose through β-glucosidic bonds. Oxidized cellulose can be either regenerated (ORC), in which organized fibers are formed prior to oxidation or non-regenerated (ONRC), with unorganized fibers priors to oxidation. Non-regenerated oxidized cellulose has frayed fibers, while regenerated oxidized cellulose has condensed fibers. ORC is thought to conform more rapidly to the surrounding environment. However, Lewis et al. [36] described superior hemostasis for ONRC, with equivalent bactericidal effectiveness, when compared to ORC. In a nonheparinized porcine hepatic square model ONRC provided superior shorter median time to hemostasis than ORC (211.2 vs 384.6 s) and at 10 min after application the hemostatic success, defined as no bleeding, was greater in the ONRC than in the ORC (100% vs 96.6%). In the same study, ONRC provided superior hemostasis in a heparinized leporine femoral bleeding model in comparison with regenerated cellulose. In particular, the hemostatic success at 90s after application was 97.5% for ONRC and 70% for ORC.

Minimal use of oxidized cellulose for effective hemostasis and maximal cleaning of field from residual agents after achievement of hemostasis is warranted. Although OC can be absorbed in 2–5 weeks, depending on the amount used, degree of saturation with blood and the tissue bed, the excess of material may also cause granulation formation without bio-degradation in the late post-operative period, and this result may complicate the radiological and clinical differential diagnosis of abscess, residual/recurrent tumors and granuloma [37, 38, 68]. Sabino et al. [37] demonstrated in a series of laparoscopic partial nephrectomies (LPN) that the use of OC was associated with an intense foreign-body reaction, strongly present at day 4,7 and 21 post-op. Tissue at 21 days in a group treated with OC was grossly solid, apparently a granuloma. Notwithstanding OC’ low pH may exert a bactericidal effect, in this study suppuration was higher in the group treated with OC, probably because the existence of residual material may serve as a bed for microorganisms in the late period, particularly in a heme-rich environment. Moreover, at day 7 post-op, the incidence of urinary fistula was higher in the presence of OC. Similarly, Agarwal [68] and coworkers have shown that after laparoscopic nephron-sparing surgery using Surgicel bolster for hemostasis, foreign-body reaction leading to the formation of pseudotumor was observed at 3 month magnetic resonance follow-up, which leads to a diagnostic dilemma.

While OC devices are designed to stimulate clot formation and to provide a favorable three-dimensional structure for clot organization, there is no evidence that they are useful in preventing biliary, pancreatic and air leaks.

Other safety considerations include potential paralysis and nerve damage when OC is used around, in or in proximity to foramina, areas of bony confine, the spinal cord, the optic nerve and chiasm [69]. Menosky et al. [69], demonstrated in a case series of decompression of high-grade spinal stenosis that the swelling of small portions of Surgicel Fibrillar, used to control bleeding from epidural venous plexus, led to a significant mass effect one day after surgery, causing a rapid neurological deterioration. Moreover, a stenotic effect when ORC has been applied as a wrap during vascular surgery or urologic procedure has been described [69]. Although it has not been established that the stenosis was directly related to the use of cellulose-based products, it is important to be cautious and avoid applying the material tightly as a wrapping.

The effective deployment of cellulose-based products may prove difficult in wet environments, as a result of poor adhesion to tissue in this setting. Frequently, the application of adequate pressure at the site of hemorrhage is required to provide the tamponade necessary to facilitate their effectiveness. For this reason, OC may be intended for use as an adjunct to gauze packing, paying attention to remove the excess material not contributing to hemostasis at reoperation, after damage control [39].

Other mechanical hemostats are represented by gelatin-based devices, prepared from purified pork skin gelatin or bovine derived gelatin. They are highly absorptive and provide a mechanical matrix for the clot to adhere. They act at the end stage of the coagulation cascade to facilitate fibrin formation, promoting coagulation and minimizing blood loss, from oozing to spurting [2]. They are available in sponge, powder and granular forms. Gelatin formulations are able to adjust to irregular wounds and surgical cavities. Because the pH of gelatin-derived devices is neutral, they can be used in conjunction with thrombin and these products belong to the category of adhesives (FloSeal® Baxter Healthcare Corporation, Fremont CA, USA; Surgiflo® Johnson & Johnson Company, NJ, USA).

Once applied, gelatin-based agents are completely reabsorbed within 4–6 weeks. Attention must be paid to use the minimal amount of agent to achieve hemostasis and to remove excess product after hemostasis has been achieved, because the swelling could result in compression and necrosis of the surrounding tissue if the product is packed or wadded, especially within bony spaces [2].

Similarly, bovine-collagen based products act by forming a physical matrix that encourages clot by stimulating platelet aggregation and the release of clotting factors.

The use of collagen-based and gelatin-based products has led to a significant reduction in both intraoperative blood loss and postoperative transfusions. The effectiveness of these two types of products has been compared in a recent randomized controlled trial performed by Xu et al. [40] on 92 patients who had undergone spinal fusion surgery. The authors concluded that collagen-based products demonstrated better hemostatic effect than gelatin-based products, with lower blood loss and postoperative drainage volume.

Caution must be paid when blood-saving devices are required during surgical procedures, because the particles of bovine-collagen based product pass through the filters [1].

Microporous polysaccharide hemospheres (MPH), a new generation hemostatic agent (Arista™ AH; Medafor Inc., Minneapolis, MN), are derived from plant starch in the form of a powder. It absorbs water and low molecular weight compound from blood to concentrate platelets and clotting proteins as its beaded surface while enhancing endogenous clotting processes [38, 41]. Its safety and efficacy have been shown in many tissues.

Bruckner et al. [41] in a retrospective study tested the product on 240 patients who had undergone cardiothoracic procedures (heart transplantation, cardiac assist devices, coronary artery bypass grafts, valve procedures, lung transplantation, aortic dissection and others). The application of Arista™ AH led to significant reduction in hemostasis time versus the untreated control group (Arista™ AH 93.4 ± 41 min vs Control 107.6 ± 56 min; *p* = 0.02). Postoperative chest drain ouptut during the first 48 h was also significantly reduced (Arista™ AH 1594 ± 949 mL vs Control 2112 ± 1437 mL, *p* < .001), as well as packed red blood cells transfusion requirements (Arista™ AH 2.4 ± 2.5 units vs Control 4.0 ± 5.1 units; *p* < .001). Emmez and coll [38] demonstrated that Arista™ AH, in comparison with ORC, provided a safe and effective hemostasis in a model of brain hemorrhage. Moreover, Arista™ AH was found to be completely re-absorbable within a couple of minute, therefore it does not appear to represent a nidus for infection or granuloma formation [41]. Safety concerns include the risk of embolism if accidentally injected or placed within blood vessels and its use on diabetic patients. In this last setting, no more than 50 g of product should be used, because amounts in excess of 50 g could affect the glucose load [41].

Table 4 summarized referral papers for topic 2.

**Table 4:** Reviewed papers for mechanical hemostats

	Year	Design	Comments	GoR-LoE
Agarwal et al. [68]	2010	Case series	Excessive amount of oxidized cellulose may induce granuloma	2C
Broadbelt et al. [39]	2002	Case series	Excessive amount of cellulose in confined space like foramina may induce compression of spinal cord because of swelling	2C
Bruckner et al. [41]	2014	Retrospective	The use of polysaccharide hemospheres absorbable hemostat in complex cardiothoracic surgery resulted in significant reduction in hemostasis time, postoperative chest tube output, and need for postoperative blood transfusion	2A
Emmez et al. [38]	2010	Experimental in vivo	Microporous polysaccharides spheres may be used for hemostasis in neurosurgical setting, without inducing granuloma	2A
Lewis et al. [36]	2013	Experimental in vivo-in vitro preclinical	Oxidized non-regenerated cellulose provides superior hemostasis and equivalent bactericidal effectiveness relative to oxidized regenerated cellulose	2C
Menovsky et al. [69]	2011	Case reports	Removal of Surgicel® Fibrillar™ is advised after hemostasis has been achieved to avoid the development of complications due to a mass effect	2C
Ragusa et al. [34]	2007	Randomized prospective	Absorbable gelatin in association with antibiotic is effective in reduce bleeding and infectious complications after cardiac surgery	2A
Sabino et al. [37]	2007	Retrospective		2B
Wagenhauser et al. [35]	2016	Experimental preclinical	Stronger inhibition of essential cellular processes of wound healing were observed for Oxidized non-regenerated cellulose when compared with oxidized regenerated cellulose	2C
Xu et al. [40]	2016	Randomized controlled	Hemostatic collagen sponge demonstrated better hemostasis effects than gelatin sponge with lower volume of postoperative drainage volume and blood loss in posterior spinal fusion	2A

### Sealants

1. *Sealants encompass a variety of heterogeneous but non-equivalent products (1A).*
2. *Sealants are effective, irrespective of patient’s coagulation status, to improve control of residual oozing (1A)*
3. *Polyethylene glycol-based sealants are effective, as an adjunctive measure, to improve hemostasis on vascular anastomosis, to control air and cerebrospinal fluid leakages (1B)*
4. *Polyethylene glycol-based sealants must not be used in cases of known or suspected allergy to human albumin or renal insufficiency (1A)*
5. *The use of cyanoacrylate-based adhesives must be avoided both in contact with brain tissue because of the risk of foreign body reaction, and inside the vessels, because of thrombotic risk, except for interventional radiology or endoscopic procedures (1A)*
6. *Weak evidences are available regarding the effectiveness of fibrin glue to prevent biliary, urinary and air leakages (1B)*
7. *Albumin-based sealants are effective to improve hemostasis on vascular anastomosis, but circumferential application must be avoided because of the risk of stenosis (1B)*
8. *Albumin-based sealants are effective to control cerebrospinal fluid leakage (1B)*

#### Scientific foundation

Sealants encompass a wide variety of products that are able to form a barrier to leakage and bleeding through covalent polymerization between themselves and adjacent tissues. These products differ one from another for the mechanism of action, field of use and warning against use. The ideal sealant should be strong, flexible, rapid to adhere, sterile, without toxicity, biologically inert, completely biocompatible, able to be used in a relatively wet environment and with low thrombogenicity.

Fibrin-based adhesives [2, 16–23] also have a sealant effect due to the polymerization of fibrin monomers. In this case the hemostatic effect is predominant, because it depends entirely on the concentration of the fibrinogen, of the factor XIII and fibronectine, whereas that of the sealant is frail. The sealant effect of fibrin glues has been investigated in a wide variety of surgical fields [2]. The most widely investigated is liver surgery because biliary leakage, although not very frequent, remains a problem with a reported incidence ranging from 3.6% up to 12% [16]. Postoperative biliary leakage may result in abdominal sepsis and even postoperative mortality. Unfortunately, results are conflicting. While some studies [16, 19] indicate that fibrin sealants may have a potential role in sealing bile ducts, thereby decreasing the total fluid drainage and its bilirubin concentration, others did not [17, 18]. In particular, Figueras et al. [17] in a randomized study on 300 patients who underwent liver resection demonstrated that the incidence of biliary fistula was similar in the fibrin glue and control groups (10% vs 11%), and that there were no differences in postoperative morbidity between the groups (23% vs 23%; *p* = 1). Similarly, scant evidence is available regarding the effectiveness of fibrin glue in preventing urinary leakage [2]. Fibrin glue has been the most widely used sealant material in thoracic surgery over the last 10 years. Moreover, several randomized trials and systematic reviews [32] demonstrated that there was no difference between treatment and control groups in terms of postoperative air leaks, duration of chest drain and in hospital length of stay, therefore discouraging its systematic use.

True sealants (“cross-linking”) can be divided into synthetic (PEG-polyethylene glycol-based and cyanoacrylate) [40–46] and semi-synthetic (glutaraldheide-albumin) [49]. In contrast to fibrin glue, cross-linking sealants polymerize through a non-enzymatic chemical reaction, which do not require the presence of blood or any components of the coagulation cascade. The presence of coagulation factors within the fibrin glue and, on the other hand, the ability of the cross-linking sealants to act even in cases of failure of the coagulation cascade, make both these products effective, irrespective of the patient’s coagulation status, as adjunctive measures to improve control of residual mild hemorrhage (oozing) [44, 46, 47].

Polyethylene-glycol-based (PEG) sealants are manufactured both as pads of different sizes (Hemopatch® Baxter AG, Vienna, Austria; Veriset™ Covidien Inc., Mansfield, MA, USA) and as flowing products (DuraSeal®, Integra LifeSciences, Plainsboro, NJ; Progel®, Neomend Inc., Irvine, CA). Available pads can be composed of a synthetic, protein-reactive monomer (NHS-PEG) and a collagen backing (Hemopatch®), or of an oxidized cellulose backing impregnated with buffer salts, trilysine and PEG derived from non-animal sources (Veriset™) [44, 46, 47]. Several studies demonstrated the effectiveness of both products as sealants and hemostats in different settings. Imkamp and coworkers [44] were the first to provide a clinical report on the effectiveness of Hemopatch® in a seven-patient case series on laparoscopic, zero-ischemia partial nephrectomy (LPN). In four of the seven patients no hemostatic under-suture was used following sealant application; in all patients the postoperative course was uneventful, and no leakages were observed. In other series, Hemopatch® was effectively used for hemostasis and sealing after coronary artery grafting, resection of the interventricular septum and aortic and mitral valve replacement while on cardiopulmonary bypass [44]. The product was used to treat the dissected myocardial tissue and the anastomotic suture lines, without any appearance in the postoperative period of pericardial effusion or pseudoaneurysm suggestive of hemostatic failure. Moreover, the effectiveness of this product depends entirely on the presence of a proteinaceous fluid to form a hydrogel, which effectively adheres and seals tissue surfaces [44], but limits its use on dry environment.

In a multicenter, randomized, single-blind clinical trial on 50 patients, Ollinger et al. [48] demonstrated the effectiveness of the oxidized cellulose-PEG-based sealant Veriset™ in achieving hemostasis after open hepatic surgery faster than the control device (median time to hemostasis:1.0 min vs 3.0 min, *p* < .001), irrespective of the size of the raw surface (bleeding sites≥100cm^2^, median time to hemostasis:1.0 min vs 4.0 min, *p* = 0.033).

Flowing products are easier to handle and effective to control air leakage during thoracic surgery and watertight closure of dural opening occurring during spine surgery. In a multicenter prospective randomized trial on 161 patients undergoing pulmonary resection, Allen and coworkers [49] demonstrated that a synthetic PEG component in a solution of human albumin (Progel®) effectively sealed intraoperative residual air leaks after standard methods (staples, sutures, or cautery) in 77% of patients compared with 16% in the control group using standard methods alone (*p* < .001). In addition, fewer patients in the Progel® group had postoperative leaks versus the control group (65% vs 85%). On the other hand, only preclinical studies are available on the efficacy on air leak control of other products, such as Hemopatch® [44]. Therefore, further studies to investigate pneumostatic ability of this device are warranted.

Dural openings occurring during spine surgery, either intentionally to access the intradural contents, or unintentionally during extradural decompression, need watertight closure whenever possible to prevent potentially significant complications (postural headache, vomiting, dizziness, photophobia, tinnitus, and pseudomeningocele). Kim and al [45], in a prospective multicenter study on 158 subjects, demonstrated that patients treated with the PEG hydrogel sealant, in addition to standard measures of dural closure, had a significantly higher rate of watertight closure than that of the controls (100%vs 64.3%, *p* < .001), without statistical differences in postoperative cerebrospinal fluid leaks, infections, and wound healing. Although neither complications nor neurological consequences related to sealant were observed in the pivotal study [45], the risk of nerve compression due to swelling of PEG after application is present. For this reason, a new low-swell PEG hydrogel sealant (Duraseal) formulation is now available. Wright and coworkers [46] recently confirmed in a prospective, 3:1 randomized, single-blind multicenter investigational study that this new low-swelling formulation is safe and effective, watertight dural closure having been immediately achieved in 100% of the study patients treated with the hydrogel. The rate of complications was comparable between spinal sealant (6.8%) and controls (8.3%), and no device-related adverse events were recorded.

Safety considerations with PEG-based sealants include the risk of impaired renal function, due to the renal clearance of the polyethylene glycol. For this reason, PEG-based sealants should not be used in cases of known renal insufficiency. PEG-based formulations containing serum albumin may induce allergic reactions in patients sensitive to human serum albumin or other device components. Furthermore, the potential risk of transmission of communicable diseases or viruses from the human serum albumin component is not completely absent.

Cyanoacrylates are synthetic sealants that rapidly polymerize in the presence of water which acts like a catalyst. Two formulations are available: octyl-2-cyanoacrylate (Dermabond, Johnson & Johnson), and N-butyl-2 cyanoacrylate (Indermil, Covidien), possibly in association with metacryloxysulpholane monomer (Glubran 2, GEM). In general, these agents are best suited for topical dermal use for the closure of skin incisions or lacerations where there is low skin tension. A systematic review of the available literature [42] did not show any difference in dehiscence, infections or satisfaction with cosmetic appearance when repairing lacerations using octyl-2-cyanoacrylate compared with standard sutures, staples or adhesive strips. If applied below the skin, foreign body reaction can occur [42]. Moreover, heavy application of sealant should be avoided because the polymerization process generates heat, inducing thermal damage. For this reason, use of the product in contact with frail tissue such as brain tissue must be discouraged. Similarly, because of the well-known risk of post-injection systemic embolization of the glue material, intravascular use of the product should be avoided. The only settings where the endovascular use of cyanoacrylate has been demonstrated to be safe are endoscopy [50] and interventional radiology [51]. Belletrutti and coworkers [50] in a retrospective analysis of 47 consecutive procedures of endoscopic gluing of bleeding varices, achieved immediate hemostasis in 93.8% of cases, with complete eradication of varices in 84% of patients. Only one case of mesenteric thrombosis was observed and the treatment failure-related mortality was 2.1%. Parildar et al. [51] in a case series of 20 patients with visceral pseudoaneurysms demonstrated that N-butyl-cyanoacrylate may be safely used to avoid proximal embolization of the visceral arteries that could not be catheterized selectively because of tortuosity, vessel size, or anatomic location. More recently, in addition to these fields of use, the application of cyanoacrylate glues has been reported for the management of urinary fistula. Selli et al. [43] described a case series of five patients suffering from iatrogenic urinary fistulas, which were successfully managed by a minimally invasive injection of N-butyl-cyanoacrylate, seemingly a valid first line treatment, justified in cases when the urinary output is not excessive and there is a favorable ratio between the length and diameter of the fistulous tract.

The sealant effect of the semisynthetic glutaraldehyde-bovine albumin based sealant (BioGlue) is achieved by a cross-linking between proteins on the surface of human tissue to those of bovine albumin. In addition, in the presence of synthetic graft materials, BioGlue adheres to them through mechanical interlocks in the interstices of the graft matrix [52]. These properties make this sealant attractive for securing and sealing vascular anastomotic sites, thus decreasing postoperative bleeding. Coselli and coworkers [52] in a randomized study on 151 patients who had undergone cardiac and vascular procedures, demonstrated that anastomotic hemostasis was achieved in 60.5% of patient treated with Bioglue compared to 39.2% (*p* = .014) of subjects treated with standard surgical repair. At the same time, patients in the BioGlue group required fewer transfusions of red blood cells and significantly fewer reinforcing pledgets on the primary anastomosis, compared with patients treated with standard sutures (*p* = .047). Caution must be used not to circumferentially apply the product around the anastomosis, because of the risk of stenosis. It seems that BioGlue led to micro pyrogranuloma, macrophage infiltrates, and fibrosis in adventitia, indicating that the glue reacts with the tissue to contribute to stenosis [52].

Notwithstanding some adverse effects have been recommended after application of BioGlue in the proximity of nerves, recent experience reported by Miscusi et al. [53] showed that BioGlue is safe and effective, even in contact with neurological structures. The authors demonstrated on 23 patients requiring dural tear repair that the use of BioGlue was effective in achieving dural watertight closure, avoiding cerebrospinal fluid leakage, with no incidence of neurological or infection-related complications.

Table 5 summarized referral papers for topic 3.

**Table 5:** Reviewed papers for Sealants

	Year	Design	Comments	GoR-LoE
Allen et al. [49]	2004	Prospective randomized	Polymeric sealants may reduce air leak after pulmonary resection	2A
Belletrutti et al. [50]	2008	Retrospective	N-butyl-2-cyanoacrylate is effective and safe for bleeding control from gastric varices	2A
Coselli et al. [52]	2003	Prospective randomized	The bovine serum albumin and glutaraldehyde sealant is a safe and effective adjunct to reduce the occurrence of anastomotic site bleeding in cardiovascular surgery	2A
Dumville et al. [42]	2014	Cochrane review	There is some evidence that dehiscence rates may be higher in wound closed with tissue adhesives than with sutures; there was no evidence of any difference between sutures and tissue adhesive for outcomes such as cosmetic appearance and satisfaction	1A
Imkamp et al. [47]	2015	Prospective	Hemopatch® is effective to improve hemostasis in nephron-sparing surgery	2B
Kim et al. [45]	2011	Prospective randomized	Polyethylene glycol hydrogel spinal sealant is effective as adjunctive measure to improve watertight closure of dura mater in spine surgery	2A
Lewis et al. [43]	2016	Systematic review	Hemopatch® is an effective, easy-to-use hemostatic agent in open and minimally invasive surgery of patients with thrombin- or platelet-induced coagulopathies	1C
Miscusi et al. [53]	2014	Prospective	Sealants are useful for dural watertight closure	2A
Ollinger et al. [48]	2013	Prospective multicenter	Polyethylene glycol patch is useful in control of bleeding from hepatic cut surface	2A
Parildar et al. [51]	2003	Prospective	N-butyl cyanoacrylate is an useful tool for angioembolization of visceral psuedoaneurism	2A
Selli et al. [43]	2013	Case reports	N-butyl-2-cyanoacrylate represent a valid first line treatment to control urinary fistula when the output is not excessive and there is a favourable ratio between length and diameter of fistula tract	2C
Wright et al. [44]	2015	Prospective, randomized controlled, multicenter	Polyethylene glycol hydrogel sealant has been proven safe and effective for provide a watertight closure as an adjunct to sutured closure of durotomies	2A

### Hemostatic dressings (mineral and polysaccharides)

1. *In a pre-hospital setting, topical hemostatic agents, minerals and polysaccharides, together with manual compression, are effective to control junctional bleeding, if tourniquets are not effective or not applicable (1B)*
2. *Minerals must be considered only for external use, because of intense exothermic reaction, and kaolin-based products should be considered as the first choice (1B)*
3. *Bandages should be preferred over granules/powders (1B)*

#### Scientific foundation

Exsanguination following severe trauma is often potentially preventable, while hemorrhage still remains an important cause of death, mostly in prehospital settings and hostile environments [54–56]. From military experience, it is well known that the three principal sites of lethal hemorrhage are truncal (67%), junctional (i.e., groin, axilla, neck) (19%) and extremities (14%) [54–62]. A report from the National Trauma Databank suggests that mortality for patients with isolated lower extremity trauma with an arterial injury is 2.8%, with a 6.6% amputation rate [55]. Although the Hartford Consensus Conference [56] encourages wider civilian use of tourniquets for the control of significant extremity hemorrhage when direct manual compression is ineffective or impractical, also supported by the American College of Surgeons Committee on Trauma [55], and notwithstanding junctional tourniquets specifically designed for a military setting are available, the management of hemorrhage in junctional areas still represents a major concern. For this reason, topical hemostatic agents have been listed as optional basic equipment for ambulances [56] for the control of significant hemorrhage in anatomic areas where tourniquets cannot be applied and where sustained direct pressure alone is not feasible.

Hemostatic agents and dressings can be classified according to their mechanism of action, the form of the agent, the delivery mechanism, and the type of wound. They are classified either as factor concentrators, procoagulants, or muco-adhesives.

Factor concentrators are mainly represented by preparations of zeolite, a microporous crystalline aluminosilicate, an inert volcanic mineral, or of smectite, a nonmetallic clay mineral composed of sodium, calcium and aluminum silicate, which acts by concentrating the cellular and protein component of blood, thereby promoting clot formation. A first-generation agent in this group was the QuikClot® (QC) granules to be poured onto the bleeding wound. A new generation product is the QuikClot Advanced Clotting Sponge® (ACS; Z-Medica; Wallingford, CT) which uses zeolite beads enclosed in a loose mesh bag, allowing for a more effective application into wound cavities and easing removal of the product during surgery. The first series of clinical experience with QC has been described by Rhee and coworkers [58], who reported the results of a survey on 103 patients, both in military and civilian settings. The overall efficacy rate was 92%, with eight cases of ineffectiveness in coagulopathic morbid patients when QC was used as a last resort. In the same series, 20 cases of intra-corporeal use (thorax, abdomen, pelvis) of the product was described, with adverse reaction represented by burns due to intense exothermic reaction and scar formation from foreign body reaction.

QuikClot was also compared to other hemostatic agents, such as chitosan-based HemCon®, (HemCon, Medical Technologies Inc., Portland, OR) in a retrospective evaluation performed by Cox et al. [64] on military patients injured after the explosion of improvised explosive devices, or gunshot wounds. Both agents were effective in the control of bleeding, but QC use was burdened by burns at the sites of application. Thermal injury resulting from zeolite use has been described also by McManus et al. [57] in a series of cases treated for major hemorrhage secondary to combat wounds. All the available data suggest that the exothermic reaction will be greater when more blood and more product is present at the site of application. For this reason, only external use of the least amount of product necessary to achieve hemostasis is warranted [57].

Procoagulant agents enhance coagulation by providing a high local concentration of coagulation factors. The paramount agent is represented by QuikClot Combat Gauze® (QCG) produced by Z-Medica (Wallingford, CT). The product is a surgical gauze coated with kaolin, an inert mineral that initiates the coagulation cascade upon contact with the injured endothelium via the contact pathway. It is not biodegradable so it must be removed from the wound before definitive repair is completed. Shina et al. [59], from the Israel Defense Forces Medical Corps, retrospectively reviewed 122 cases of military patients on whom 133 hemostatic dressings were applied. Dressings were used in 27.8% for junctional hemorrhage control, achieving hemostasis in 88.6% of cases. For extremity injuries, hemorrhage control was achieved in 91.9% of patients. These results suggest that a kaolin-based product is an effective tool for hemorrhage control for both junctional and non-junctional injuries, with the advantage over QC of not inducing a temperature increase in the wound.

Muco-adhesive agents are represented by chitosan-based products HemCon®, (Medical Technologies Inc., Portland, OR); HemCon ChitoGauze®, (HemCon Medical Technologies Inc); Celox®, (MedTrade Products, UK). Chitosan refers to a series of polymers derived from crustacean chitin and it is a complex carbohydrate that is biodegradable. Chitosan has no intrinsic hemostatic properties and thus works independently of the coagulation system. The hemostatic properties appear to be by direct electrostatic interaction between negatively charged cell membranes of the erythrocytes and positively charged chitosan. This agent displays strong adherence to tissues and physically seals bleeding wounds [60–63]. Sixty-four combat uses of chitosan-based product were reported by Wedmore et al. [60], in a retrospective analysis of prehospital combat casualties. In this series, dressings were utilized externally on the chest, groin, buttock and abdomen in 25 cases; on extremities in 35 cases; and on neck and facial wounds in 4 cases. In 66% of patients, dressings were utilized following gauze failure and were 100% successful. In 97% of cases their use resulted in cessation of bleeding or improvement of hemostasis. Failure occurred in only two cases, because of blind application of bandages up into large cavitational injuries. The largest prospective study in a civilian setting about the use of chitosan-based hemostatic devices (HemCon ChitoGauze®) was made by Te Grotenhuis and coll [63]. They analyzed 66 cases of patients on whom dressings were applied if conventional treatment (gauze with manual compression) failed to control external bleeding or if conventional treatment was unlikely to achieve hemostasis. Twenty-one patients were taking anticoagulants or suffered from a clotting disorder. In this study, HemCon ChitoGauze® completely stopped hemorrhage in 70% and reduced hemorrhage in 20% of the patients. The cessation of hemorrhage was observed more often in patients with arterial hemorrhage (*p* = 0.0031). The result was not affected by coagulopathy and no adverse effects were recorded.

A safety concern pertains to the risk of adverse events induced by the use of chitosan-based devices in patients allergic to crustaceans. Waibel et al. [61] in 2011 tested for the first time the safety of HemCon® bandage in shellfish allergic subjects. Nineteen patients were recruited, all of whom demonstrated shrimp-specific IgE, but none had a skin prick test (SPT) positive to chitosan powder or experienced an adverse reaction during bandage challenges. No other studies using chitosan bandages have reported any allergic response to the agent. Nevertheless, although multiple reports on the use of chitosan in clinical settings exist, evidence for the safety of chitosan dressing is encouraging but not conclusive [55].

Taking into account all the aforementioned aspects regarding the different agents, mostly pertaining to potential adverse effects associated with their use, and whereas tourniquet application should be the initial and principal method for extremity hemorrhage control, kaolin-based QCG may be considered as the initial tool for junctional zone hemorrhage control.

Undoubtedly the treatment and coverage of a complex and irregular wound with multiple bleeding sites is easier when granular agents are used, however granular materials are more difficult to handle and apply effectively, especially in hostile conditions [62]. In addition, the majority of granular agents are not bio-absorbable and therefore must be removed from wounds by saline flushes or tissue debridement before surgical repair can be performed.

Moreover, as Kheirabadi and coworkers demonstrated in a preclinical study [62], there is a potential risk for some small particles to enter the vascular system and become a source of thrombosis upon blood reflow. For these reasons, bandage/gauze format that allows packing of the wound, should be preferred as being safer and able to induce a superior hemorrhage control [55].

Table 6 summarized the referral papers for topic 4.

**Table 6:** Reviewed papers for Hemostatics Dressing

	Year	Design	Comments	GoR-LoE
Bulger et al. [55]	2014	Guidelines	Topical hemostatic agent in combination with direct pressure are effective for bleeding control in pre-hospital settings in anatomical areas where tourniquet cannot be applied	1A
Cox et al. [64]	2009	Retrospective cohort study	HemCon appears to be safe, while QuickClot may produce superficial burns. These products should be taken into account to assist in controlling internal hemorrhage, especially during damage control surgery	2C
Jacobs et al. [56]	2013	Guidelines	Wider civilian use of tourniquet should be encouraged for the control of significant extremity hemorrhage when direct manual compression is ineffective or impractical	1A
Kheirabadi et al. [62]	2009	Experimental, animal	The hemostatic agents are more effective and safe than the currently deployed devices in controlling arterial hemorrhage	2A
Leonard et al. [54]	2016	Retrospective, multicenter	QuickClot is an effective and safe adjunct to control hemorrhage in prehospital setting	2A
McManus [57]	2007	Case series	Zeolite, despite potential complication of thermal injuries, has shown to be a valuable hemostatic agent if used under appropriated circumstances	2C
Rhee et al. [58]	2008	Case series	QuickClot has been proven safe and effective to control prehospital non compressible hemorrhage	2A
Shina et al. [59]	2015	Retrospective	Hemostatic dressing seem to be an effective tool for junctional hemorrhage control and should be considered as second-line treatment for extremity hemorrhage control at the point of injury	1C
Te Grotenhuis et al. [63]	2016	Prospective	Chitogauze is an effective and safe adjunct in the prehospital traatment of massive external traumatic hemorrhage	1C
Waibel et al. [61]	2011	Prospective	Chitosan bandage is safe even in shellfish allergic patients	2C
Wedmore et al. [60]	2006	Retrospective	Chitosan-based hemostatic dressing is useful for prehospital combat casualties	2C

*ORC* oxidized regenerated cellulose, *ONRC* oxidized non-regenerated cellulose

## Conclusions

In conclusion, local hemostatic agents are different products with distinctive indications. A knowledge of the properties of each single agent should be in the armamentarium of acute care surgeons in order to select the appropriate product in different clinical conditions. The aim should be to stop the bleeding as soon as possible, to avoid the consequences of prolonged blood loss on body physiology.

**Fig. 2:**
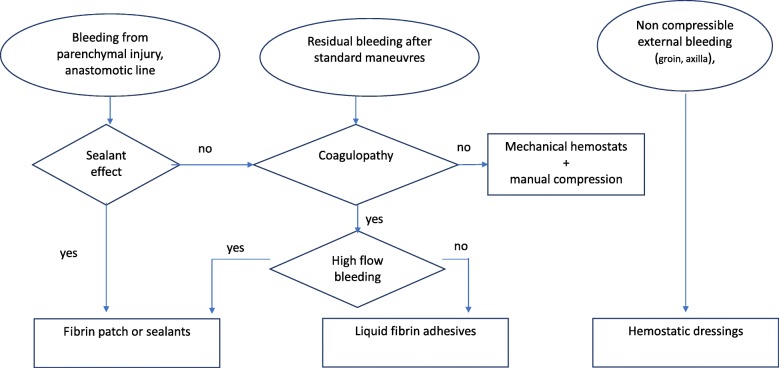
Decisional pathway to choose topical hemostats. This figure represents criteria to be applied in order to correctly select the topical hemostats according to clinical setting and patient characteristcs

The first concern (Fig. 2) of the surgeon should be the state of the endogenous coagulation system of the patient. If it is normal, a mechanical agent as adjunct to surgical hemostasis or packing is sufficient and cost-effective. If coagulation cascade does not work, in relation to the current disease or drug-induced dysfunction, the choice should be an agent that is also effective without coagulation factors, such as adhesive products. In the case of ongoing arterial or high flow bleeding, a patch-supplemented adhesive agent is easier to use because of its direct applicability under pressure on the site of the blood loss. When the bleeding source is a parenchymal section (liver, pancreas, kidney) or the objective is an airtight closure of a lung wound, the choice of a sealant agent is a valuable option. Finally, in pre-hospital settings, hemostatic dressings should be considered in junctional and non-compressible hemorrhages (neck, groin, axilla), kaolin-based products being the first choice.

From this systematic review it is clear that in acute care surgical services all categories of agents, mechanical, adhesives, adhesives with patch and sealants should be available because of their different usages and indications. On ground and air ambulances, as well as tourniquets and conventional bandages, advanced hemostatic dressings should be available to stop the bleeding in the field in complex situations.

## Abbreviations

CF: Collagen fleece
CSF: Cerebro spinal fluid
DP: Distal pancreatectomy
LPN: Laparoscopic partial nephrectomies
MPH: Microporous polysaccharide hemospheres
NCM: National Consensus Meeting
NPE: National panel of experts
OC: Oxidized cellulose
ONRC: Oxidized non-regenerated cellulose
ORC: Oxidized regenerated cellulose
PEG: Polyethylene glycol
PG: Polyglactin
POPF: Postoperative pancreatic fistula
PTFE: Polytetrafluoroethylene
QC: QuickClot®
QC-ACS: QuickClot Advanced Clotting Sponge®
QCG: QuickClot Combat Gauze®
SB: Scientific board
SICUT: Italian Society of Emergency Surgery and Trauma
TSG: Thrombin-soaked gelatin sponge
WSES: World Society of Emergency Surgery

## References

[1] Dhillon S (2011). Fibrin sealants (Evicel®, [Quixil®/Crosseal™]) a review of its use as supportive treatment for haemostasis in surgery. Drugs.

[2] Echave M, Oyaguez I, Casado MA (2014). Use of Floseal®, a human gelatin-thrombin matrix sealant, in surgery: a systematic review. BMC Surg.

[3] Chalmers RTA, Darling RC, Wingard JT, Chetter I, Cutler B, Kern JA (2010). Randomized clinical trial of tranexamic acid-free fibrin sealant during vascular surgical procedures. Br J Surg.

[4] Chapman WC, Singla N, Genyk Y, McNeil JW, Renkens KL, Reynolds TC (2015). A phase 3, randomized, double-blind comparative study of the efficacy and safety of topical recombinant human thrombin and bovine thrombin in surgical hemostasis. J Am Coll Surg.

[5] Fischer CP, Wood CG, Shen J, Batiller J, Hart JC, Patel B (2011). A randomized trial of aprotinin-free fibrin sealant versus absorbable hemostat. Clin Appl Thromb Hemost.

[6] Schwartz M, Madariaga J, Hirose R, Shaver T, Sher L, Chari R, et al. Comparison of a new fibrin sealant with standard topical hemostatic agents. Arch Surg. 2004; 139(11):1148–154. 10.1001/archsurg.139.11.1148 15545559

[7] Spotnitz WD (2014). Fibrin sealant: the only approved hemostat, sealant, and adhesive- a laboratory and clinical perspective. Surgery.

[8] Taylor ML, Mueller-Velten G, Koslow A, Hunter G, Naslund T, Kline R (2003). Prospective randomized multicenter trial of fibrin sealant versus thrombin-soaked gelatin sponge for suture- or needle-hole bleeding from polytetrafluoroethylene femoral artery grafts. J Vasc Surg.

[9] Saha SP, Muluk S, Schenk W, Dennis JW, Ploder B, Grigorian A (2012). A prospective randomized study comparing fibrin sealant to manual compression for the treatment of anastomotic suture-hole bleeding in expanded polytetrafluoroethylene grafts. J Vasc Surg.

[10] Fischer CP, Bochicchio G, Shen J, Patel B, Batiller J, Hart JC (2013). A prospective, randomized, controlled trial of the efficacy and safety of fibrin pad as an adjunct to control soft tissue bleeding during abdominal, retroperitoneal, pelvic, and thoracic surgery. J Am Coll Surg.

[11] Koea JK, Baldwin P, Shen J, Patel B, Batiller J, Arnaud A (2015). Safety and hemostatic effectiveness of the fibrin pad for severe soft-tissue bleeding during abdominal retroperitoneal, pelvic, and thoracic (non-cardiac) surgery: a randomized, controlled, superiority trial. World J Surg.

[12] Genyk Y, Kato T, Pomposelli JJ, Wright JK, Sher LS, Tetens V (2016). Fibrin sealant patch (TachoSil) vs oxidized regenerated cellulose patch (surgical original) for the secondary treatment of local bleeding in patients undergoing hepatic resection: a randomized controlled trial. J Am Coll Surg.

[13] Maisano F, Kjaergard HK, Bauernschmitt R, Pavie A, Rabago G, Laskar M (2009). TachoSil patch versus conventional haemostatic fleece material for control of bleeding in cardiovascular surgery: a randomized controlled trial. Eur J Cardio-thoracic surgery.

[14] Colombo GL, Bettoni D, Di Matteo S, Grumi C, Molon C, Spinelli D (2014). Economic and outcomes consequences of TachoSil®: a systematic review. Vasc Health Risk Manag.

[15] Simo KA, Hanna EM, Imagawa DK, Iannitti DA (2012). Hemostatic agents in hepatobiliary and pancreas surgery: a review of the literature and critical evaluation of a novel carrier-bound fibrin sealant (TachoSil). Surgery.

[16] Erdogan D, Busch ORC, Gouma DJ, van Guilk TM (2007). Prevention of biliary leakage after partial liver resection using topical hemostatic agents. Dig Surg.

[17] Figueras J, Llado L, Miro M, Ramos E, Torras J, Fabregat J (2007). Application of fibrin glue sealant after hepatectomy does not seem justified. Ann Surg.

[18] Kobayashi S, Takeda Y, Nakahira S, Tsujie M, Shimizu J, Miyamoto A (2016). Fibrin sealant with polyglycolic acid felt vs fibrinogen-based collagen fleece at the liver cut surface for prevention of postoperative bile leakage and hemorrhage: a prospective, randomized, controlled study. J Am Coll Surg.

[19] De Boer MT, Boonstra EA, Lisman T, Porte RJ (2012). Role of fibrin sealants in liver surgery. Dig Surg.

[20] Perussi Biscola N, Cartarozzi LP, Ulian-Benitez S, Barbizan R, Castro MV, Barroso Spejo A (2017). Multiple use of fibrin sealant for nervous system treatment following injury and disease. J Venom Anim Toxins Incl Trop Dis.

[21] Jankowitz BT, Atteberry DS, Gerszten PC, Karausky P, Cheng BC, Faught R (2009). Effect of fibrin glue on the prevention of persistent cerebrospinal fluid leakage after incidental durotomy during lumbar spinal surgery. Eur Spine J.

[22] Green AL, Arnaud A, Batiller J, Eljamel S, Gauld J, Jones P (2015). A multicenter prospective, randomized, controlled study to evaluate the use of a fibrin sealant as an adjunct to sutured dural repair. Br J Neurosurg.

[23] Esposito F, Angileri FF, Kruse P, Cavallo LM, Solari D, Esposito V, et al. Fibrin sealants in dura sealing: a systematic literature review. PLOS ONE. 2016; 12(4):e0175619. 10.1371/journal.pone.0151533 PMC4847933 27119993

[24] Briceno J, Naranjo A, Ciria R (2010). A prospective study of the efficacy of clinical application of a new carrier-bound fibrin sealant after liver resection. Arch Surg.

[25] Vakalopoulos KA, Daams F, Wu TZ (2013). Tissue adhesives in gastrointestinal anastomosis: a systematic review. Journal of Surgical Res.

[26] Pommergaard HC, Achiam MP, Rosemberg I (2012). External coating of colonic anastomoses: a systematic review. Interational Journal of Colorectal Diseases.

[27] Montorsi M, Zerbi A, Bassi C, Capussotti L, Coppola R, Sacchi M (2012). Efficacy of an absorbable fibrin sealant patch (TachoSil) after distal pancreatectomy. Ann Surg.

[28] Pavlik Marangos I, Rososk BI, Kazaryan AM, Rosseland AR, Edwin B (2011). Effect of TachoSil patch in prevention of postoperative pancreatic fistula. J Gastrointest Surg.

[29] Cheng Y, Ye M, Xiong X, Peng S, Wu HM, Cheng N (2016). Fibrin sealants for the prevention of postoperative pancreatic fistula following pancreatic surgery (Review). Cochrane Database of Systematic Reviews.

[30] Filosso PL, Ruffini E, Sandri A, Lausi PO, Giobbe R, Oliaro A (2013). Efficacy and safety of human fibrinogen-thrombin patch (TachoSil®) in the treatment of postoperative air leakage in patients submitted to redo surgery for lung malignancies: a randomized trial. Interact Cardiovasc Thorac Surg.

[31] Anegg U, Lindenmann J, Matzi V, Smolle J, Maier A, Smolle-Juttner F (2007). Efficeincy of fleece-bound sealing (TachoSil®) of air leaks in lung surgery: a prospective randomised trial. Eur J Cardiothorac Surg.

[32] Belda-Sanchis J, Serra-Mitjans M, Iglesias Sentis M, Rami R (2010). Surgical sealant for preventing air leaks after pulmonary resections in patients with lung cancer (Review). Cochrane Database of Systematic Reviews.

[33] Cormio L, Perrone A, Di Fino G. TachoSil® sealed tubeless percutaneous nephrolithotomy to reduce urine leakage and bleeding: outcome of a randomized controlled trial. J Urol. 2012; 188(1):145–50. 10.1016/j.juro.2012.03.011 22591964

[34] Ragusa R, Faggian G, Rungatscher A, Cugola D, Macron A, Mazzucco A (2007). Use of gelatin powder added to rifamycin versus bone wax in sternal wound hemostasis after cardiac surgery. Interact Cardiovasc Thorac Surg.

[35] Wagenhauser MU, Mulorz J, Simon F, Spin JM, Schelzig H, Oberhuber A (2016). Oxidized (non)-regenerated cellulose affects fundamental cellular processes of wound healing. Scientific Reports.

[36] Lewis KM, Spazierer D, Urban MD, Lin L, Redl H, Goppelt A (2013). Comparison of regenerated and non-regenerated oxidized cellulose hemostatic agents. Eur Surg.

[37] Sabino L, Andreoni C, Faria EF, Ferreira PSVS, Paz AR, Kalil W (2007). Evaluation of renal defect healing, hemostasis, and urinary fistula after laparoscopic partial nephrectomy with oxidized cellulose. J Endourol.

[38] Emmez H, Tonge M, Tokgoz N, Durdag N, Gonul I, Ceviker N (2010). Radiological and Histopathological Comparison of Microporous Polysaccharide Hemospheres and Oxidized regenerated Cellulose in the rabbit Brain: A Study of Efficacy and Safety. Turkish Neurosurgery.

[39] Brodbelt AR, Miles JB, Foy PM, Broome JC (2002). Intraspinal oxidized cellulose (Surgicel) caused a delayed paraplegia after thoracotomy-a report of three cases. Ann R Coll Surg Engl.

[40] Xu D, Ren Z, Chen X, Zhuang Q, Sheng L, Shugang L (2016). A randomized controlled trial on effects of different hemostatic sponges in posterior spinal fusion surgeries. BMC Surg.

[41] Bruckner B, Blau LN, Rodriguez L, Suarez EE, Ngo UQ, Reardon MJ (2014). Microporous polysaccharide hemosphere absorbable hemostat use in cardiothoracic surgical procedures. J Cardiothoracic Surg.

[42] Dumville JC, Coulthard P, Worthington HV, Riley P, Patel N, Darcey J (2014). Tissue adhesives for closure of surgical incisions (Review). Cochrane Database of Systematic Reviews.

[43] Selli C, De Maria M, Manica M, Turri FM, Manassero F (2013). Minimally invasive treatment of urinary fistulas using N-Butyl-2-cyanoacrylate: a valid first line option. BMC Urol.

[44] Lewis KM, Kuntze CE, Gulle H (2016). Control of bleeding in surgical procedures: critical appraisal of HEMOPATCH (sealing hemostat). Medical Devices: Evidence and Research.

[45] Kim KD, Wright NM (2011). Polyethylene glycol hydrogel spinal sealant (DuraSeal spinal sealant) as an adjunct to sutured dural repair in the spine: results of a prospective multicenter, randomized controlled study. Spine.

[46] Wright NM, Park J, Tew JM, Kim KD, Shaffrey ME, Cheng J (2015). Spinal sealant system provides better intraoperative watertight closure than standard of care during spinal surgery. Spine.

[47] Imkamp F, Tolkach Y, Wolters M, Jutzi S, Kramer M, Herrmann T (2015). Initial experience with the Hemopatch® as a hemostatic agent in zero-ischemia partial nephrectomy. World J Urol.

[48] Ollinger R, Mihaljevic AL, Schuhmacher C, Bektas H, Vondran F, Kleine M (2013). A multicenter, randomized clinical trial comparing Veriset™ haemostatic patch with fibrin sealant for the management of bleeding during hepatic surgery. HPB.

[49] Allen MS, Wood DE, Hawkinson RW (2004). Prospective randomized study evaluating a biodegradable polymeric sealant for sealing intraoperative air leaks that occur during pulmonary resection. Ann Thorac Surg.

[50] Belletrutti PJ, Romagnuolo J, Hilsden RJ, Chen F, Kaplan B, Love J (2008). Endoscopic management of gastric varices: efficacy and outcomes of gluing with N-butyl-2-cyanoacrylate in a north American patient population. Can J Gastroenterol.

[51] Parildar M, Oran I, Memis A (2003). Embolization of visceral pseudoaneurysms with platinum coils and N-butyl cyanoacrylate. Abdom Imaging.

[52] Coselli JS, Bavaria JE, Fehrenbacher J, Stowe CL, Macheers SK, Gundry SR (2003). Prospective randomized study of a protein-based tissue adhesive used as hemostatic and structural adjunct in cardiac and vascular anastomotic repair procedures. J Am Coll Surg.

[53] Miscusi M, Polli FM, Forcato S, Coman SA, Ricciardi L, Ramieri A (2014). The use of surgical sealants in the repair of dural tears during non-instrumented spinal surgery. Eur Spine J.

[54] Leonard J, Zietlow J, Morris D, Berns K, Eyer S, Martinson K (2016). A multi-institutional study of hemostatic gauze and tourniquets in rural civilian trauma. J Trauma Acute Care Surg.

[55] Bulger EM, Snyder D, Schoelles K, Gotschall C, Dawson D, Lang E (2014). An evidence-based prehospital guideline for external hemorrhage control: American College of Surgeons Committee on trauma. Prehospital Emergency Care.

[56] Jacobs LM, McSwain NE, Rotondo MF, Wade D, Fabbri W, Eastman A (2013). Joint committee to create a national policy to enhance survivability from mass casualty shooting events. Improving survival from active shooter events: the Hartford consensus. J Trauma Acute Care Surg.

[57] McManus J, Hurtado T, Pusateri A, Knoop KJ (2007). A case series describing thermal injury resulting from zeolite use for external hemorrhage control in combat operations. Prehospital Emergency care.

[58] Rhee P, Brown C, Martin M, Salim A, Plurad D, Green D (2008). QuickClot use in trauma for hemorrhage control: case series of 103 documented uses. J Trauma.

[59] Shina A, Lipsky AM, Nadler R, Levi M, Benov A, Ran Y (2015). Prehospital use of hemostatic dressings by the Israel defense forces medical corps: a case series of 122 patients. J Trauma Acute Care Surg.

[60] Wedmore I, McManus JG, Pusateri AE, Holcomb JB (2006). A special report on the chitosan-based hemostatic dressing: experience in current combat operations. J Trauma.

[61] Waibel KH, Haney B, Moore M, Whisman B, Gomez R (2011). Safety of chitosan bandages in shellfish allergic patients. Mil Med.

[62] Kheirabadi BS, Edens JW, Terrazas IB, Estep JS, Klemcke HG, Dubick MA (2009). Comparison of new hemostatic granules/powders with currently deployed hemostatic products in a lethal model of extremity arterial hemorrhage in swine. J Trauma.

[63] Te Grotenhuis R, van Grunsven PM, Heutz WMJM, Tan ECTH (2016). Prehospital use of haemostatic dressings in emergency medical services in the Netherlands: a prospective study of 66 cases. Injury.

[64] Cox ED, Schreiber MA, McManus J, Wade JC, Holcomb JB (2009). New hemostatic agents in the combat setting. Transfusion.

[65] National Institute of Health, Consensus Development Program, 2013. Available at https://consensus.nih.gov

[66] Moher D, Liberati A, Tetzlaff J, Mulrow C, Gotzsche PC, Ioannidis JP, Clarke M, Devereaux PJ, Kleijnen J, Moher D (2009). Preferred reporting items for systematic reviews and meta-analyses. The PRISMA Statement. PLoS Med.

[67] Guyatt G, Gutterman D, Baumann MH, Addrizzo-Harris D, Hylek EM, Phillips B, Raskob G, Lewis SZ, Schunemann H (2006). Grading strength of recommendations and quality of evidence in clinical guidelines: report from an American College of Chest Physicians task force. Chest.

[68] Agarwal MM, Mandal AK, Agarwal S, Lal A, Prakash M, Mavuduru R (2010). Surgicel granuloma: unusual cause of “recurrent” mass lesion after laparoscopic nephron-sparing surgery for renal cell carcinoma. Urology.

[69] Menovsky I, Plazier M, Rasschaert R, Maas AIR, Parizel PM, Verbeke S (2011). Massive swelling of Surgicel® Fibrillar™ hemostat after spinal surgery. Case reports and a review of the literature. Minim Invasive Neurosurg.

